# Eight gene mutation-based polygenic hazard score as a potential predictor for immune checkpoint inhibitor therapy outcome in metastatic melanoma

**DOI:** 10.3389/fmolb.2022.1001792

**Published:** 2022-09-02

**Authors:** Liqin Zhao, Ting Luo, Jinling Jiang, Junwei Wu, Xiaowei Zhang

**Affiliations:** ^1^ Department of Oncology, Ruijin Hospital, Shanghai Jiao Tong University School of Medicine, Shanghai, China; ^2^ Department of Gastrointestinal Medical Oncology, Fudan University Shanghai Cancer Center, Shanghai, China; ^3^ Department of Oncology, Shanghai Medical College, Fudan University, Shanghai, China; ^4^ Department of Oncology, Chengdu First People’s Hospital, Chengdu, China

**Keywords:** immune checkpoint inhibitor, somatic gene mutation, polygenic hazard score, metastatic melanoma, tumor mutation burden

## Abstract

**Background:** Immune checkpoint inhibitor (ICI) therapies have revolutionized the treatment of metastatic cutaneous melanoma, but have only benefitted a subset of them. Gene mutations were reported to impact the ICI therapy outcomes in metastatic melanoma but have not been fully investigated. Hence, we systematically analyzed the impact of cancer-related gene mutations on the clinical outcome in metastatic melanoma patients who underwent ICI therapies.

**Methods:** Publicly available discovery and validation cohorts (312 patients and 110 patients respectively, all the patients received ICI therapies) were included in this study. Cox proportional hazards regression analysis was used to assess the association of 468 cancer-related gene mutations with overall survival (OS) in the discovery cohort, and the polygenic hazard score (PHS) was constructed subsequently, and validated in the validation cohort. The Tumor Immune Estimation Resource (TIMER) online tools, which are based on The Cancer Genome Atlas database, were used to analyze the impact of gene mutations on tumor-infiltrated immune cells in melanoma samples.

**Results:** We found eight gene mutations that were significantly associated with the overall survival (*BAP1, CARD11, IGF1R, KMT2D, PTPRD, PTPRT, ROS1, and TERT*, *p* < 0.05, mutation frequency >0.05). The PHS, which was based on these genes, was found to effectively discriminate the subset which benefited most from ICI therapies (HR = 1·54, 95%CI, 1.25–1.95; *p* < 0.001). After adjusting with age, sex, ICI regimes, and tumor mutation burden (TMB), we found that PHS was an independent predictor for the outcome of ICI therapies (adjusted HR = 1.84, 95%CI, 1.22–2.79; *p* = 0.004). The PHS was validated in the validation cohort (log-Rank *p* = 0.038). Further research found that *CARD11* and *PTPRD* mutations were significantly associated with more tumor-infiltrated immune cells in melanoma samples.

**Conclusion:** For the first time, we have shown that PHS can independently and effectively predict the ICI therapy outcome in metastatic melanoma, which once validated by larger research, may help the decision-making process in melanoma.

## Introduction

Melanoma is notorious for its rapid progression and enormous capacity of metastasis. In 2018, over 280,000 new patients suffered from cutaneous melanoma with over 60,000 cancer-related deaths worldwide (Bray et al., 2018). In Asia, the mucosal and acral melanomas accounted for up to 58% of all melanoma types and were more aggressive compared with cutaneous melanoma (Chang et al., 2004).

Because of its intrinsic property of resistance to chemo/radiotherapy, the mainstay therapies for metastatic melanoma have been limited to high-dose interleukin-2, dacarbazine, and vemurafenib before the emergence of immune checkpoint inhibitors (ICI), (Flaherty et al., 2010), and the 5-year survival rate stayed between 15–20 % for decades, which was quite unsatisfactory (Siegel et al., 2018). The ICI treatments, including antibodies targeting programmed death-1 (PD-1), programmed death-ligand 1 (PD-L1), or cytotoxic T-lymphocyte-associated protein 4 (CTLA4), provide substantial survival benefits for metastatic melanoma (Hodi et al., 2010) (Hodi et al., 2018), especially with durable anti-tumor effects for patients who achieved complete response after receiving ICI therapies, with over 85 % having a 5-year progression-free survival rate; while for unselected patients, the 5-year survival rate was 43.2 % and 33.0 % for anti-PD-1 therapy and anti- CTLA4, respectively, according to the KEYNOTE-006 research (Robert et al., 2019). For the acral and mucosal melanomas, the pembrolizumab, an anti-PD-1 monoclonal antibody, had achieved limited efficacy in second line settings, with ORR 15.8 % and 13.3 %, respectively (Si et al., 2019). Hence, it is critical to identify sensitive predictors for the ICI outcome in metastatic melanoma.

Previous studies showed that the PD-L1 expression level was not a solid predictor for ICI therapy (Carlino et al., 2018) (Larkin et al., 2015). Tumor mutation burden (TMB) was validated to be an effective predictor for ICI treatment outcome in various cancer types, as high TMB tumors might yield more neoantigens, which were believed to trigger the tumor immunity, but this association was not significant in metastatic melanoma (Samstein et al., 2019). Recently, some research studies reported that somatic gene mutations were associated with the ICI therapy outcomes in various cancer types including metastatic melanoma (Wang et al., 2019) (Xiao et al., 2018). However, it was believed that a single somatic gene mutation was not efficient enough to detect the beneficial metastatic melanoma subgroups for ICI therapies. As reported, the 21 gene model in breast cancer prognostic prediction could effectively assess the recurrence risk and help treatment decision-making (Varga et al., 2019); it was reasonable to speculate that the polygenic mutation model might be effective to predict the ICI therapy outcomes. Hence, we systematically analyzed the association of 468 cancer-related gene mutations with the ICI therapy outcomes in a melanoma cohort consisting of 321 patients, constructed a polygenic hazard score (PHS), and validated our findings in another independent cohort, in this post-hoc analysis.

## Materials and methods

### Data acquisition

Two independent datasets were downloaded from the publicly available cBioPortal database (https://www.cbioportal.org), i.e., the Memorial Sloan Kettering Cancer Center Integrated Mutation Profiling of Actionable Cancer Targets (MSK-IMPACT) dataset (https://www.cbioportal.org/study/summary?id=tmb_mskcc_2018), which included 321 metastatic melanoma patients who received anti-PD-1/PD-L1 therapy, anti-CTLA-4 therapy or a combination of anti-PD-1/PD-L1 with anti-CTLA-4 therapies (Samstein et al., 2019); and the DFCI (Dana-Farber Cancer Institute) metastatic melanoma (DFCIMM) dataset (https://www.cbioportal.org/study/clinicalData?id=skcm_dfci_2015) which included 110 metastatic melanoma patients who received anti-CTLA-4 therapy alone (Van Allen et al., 2015). The MSK-IMPACT dataset contained 468 cancer-related gene-sequencing data, and the detailed gene list is included in Supplementary Table S1. DFCIMM datasets contained whole exome sequencing data. Both of them contained integral clinical and survival information.

### Discovery analysis

We used the MSK-IMPACT dataset consisting of all somatic mutation data, except the synonymous mutation of the 468 cancer-related genes, to assess which gene mutation was significantly associated with the overall survival (OS). We divided the 321 metastatic melanoma patients into two groups according to a certain gene mutational status: the group of patients with certain gene mutations, and the other group of patients carrying the gene without mutations or with synonymous mutation (e.g., the *TP53* mutational group and the *TP53* wildtype or synonymous mutational group), and then we performed the univariate Cox proportional hazards regression analysis for these two groups to evaluate the association between gene mutation status and the OS of metastatic melanoma patients who received ICI therapies. Each of the 468 genes was assessed accordingly, and those genes with *p* value < 0.05 and mutation frequency >0.05 (i.e., mutation counts >321 × 5%) were selected as candidate genes for the multivariate Cox regression analysis with adjustments for age, sex, TMB, and ICI regimes (i.e., anti-PD-1/PD-L1, anti-CTLA-4, and the combination of anti-PD-1/PD-L1 with anti-CTLA-4). Kaplan–Meier survival curves were generated to visualize the results.

### Construct the polygenic hazard score model

The PHS was built and used as an effective survival analysis model to predict the time to events (Tan et al., 2019) (Seibert et al., 2018). Those genes with *p* value < 0.05 and mutation rate >0.05 were used to construct the PHS model. The score was defined as the vector product of a patient’s mutational status (MS) (MS = 1 or 0, MS = 1 represents the patients carrying the mutational gene, MS = 0 represents the patients carrying the wildtype gene or synonymous gene) and the corresponding log hazard ratio (logHR) from the aforementioned Cox proportional hazards regression for the selected genes (see equation).

$$PHS=\sum_{n=1}^{m}logHR_{n}\times MS_{n}$$ (1)

To ascertain whether the PHS was independently associated with OS or not, we subsequently performed the multivariate Cox regression analysis of PHS with adjustments for age, sex, TMB, and ICI regimes, and divided the 321 metastatic patients into three different levels (high-, intermediate-, and low-risk groups) according the PHS value, finally generating the Kaplan–Meier survival curves.

### Validation analysis

We further validated this PHS mode in the DFCIMM cohort using the calculation method mentioned previously. Then, the 110 metastatic patients were also divided into three different groups according the PHS value, and Kaplan–Meier survival curves were generated at last.

### Tumor-infiltrated immune cells’ assessment

In order to explore the possible mechanisms underlining the impact of those gene mutations on the survival of metastatic melanoma who received ICI therapies, we compared the differences in tumor-infiltrated immune cells between the groups of the patients carrying mutational genes and patients carrying wildtype genes from the (TCGA) dataset using TIMER online tools (https://cistrome.shinyapps.io/timer/) (Li et al., 2017), and boxplots were obtained.

All the data were analyzed by using the R language (version 3.5.1), and *p* values were two-sided with a significance level of 0.05.

## Results

### Eight genes were found in the discovery analysis

A total of 321 metastatic melanoma patients from the MSK-IMPCAT cohort who received immunotherapies were analyzed (116 patients received a combination of anti CTLA-4 and anti PD-1/PD-L1 immunotherapies, 75 and 130 patients received anti CTLA-4 and anti PD-1/PD-L1 respectively), and the general clinicopathological characteristics of these patients are presented in Table1. Meanwhile, clinical information of patients in the validation cohort (DFCIMM) is presented in Supplementary Table S2.

**TABLE 1:** Clinicopathological characteristics of the MSK-IMPACT metastatic melanoma cohort that underwent ICI treatment.

Characteristics	No. of cases (%)
All subjects	321 (100)
Age at diagnosis (year)	
<30	15 (4.7)
31–50	52 (16.2)
50–60	73 (22.7)
61–70	85 (26.5)
>71	96 (29.9)
Sex	
Female	121 (37.7)
Male	200 (62.3)
ICI regime	
Combo	116 (36.1)
CTLA4	75 (23.4)
PD-1/PDL-1	130 (40.5)
Mean of TMB (/Mb) ± SD	18.60 ± 24.78

MSK-IMPACT, memorial sloan kettering cancer center integrated mutation profiling of actionable cancer targets; ICI, immune checkpoint inhibitors; Combo, anti CTLA-4, combined with anti PD-1/PD-L1; TMB, tumor mutation burden;/Mb, per Mega bases; SD, standard deviation.

In the univariate Cox regression analysis, eight gene mutations were found to be significantly associated with OS in this cohort, i.e., *BAP1, CARD11, IGF1R, KMT2D, PTPRD, PTPRT, ROS1,*and *TERT* (HR = 1.72, 0.58, 0.47, 0.62, 0.66, 0.71, 0.68, and 0.71 respectively; *p* = 0.028, 0.033, 0.034, 0.034, 0.029, 0.029, 0.043, and 0.008, respectively)*;* details are included In Table 2.

**TABLE 2:** These gene mutations were significantly associated with overall survival in the MSK-IMPACT metastatic melanoma cohort.

Gene symbol	HR	95% CI	Mutation counts	*p* value
*BAP1*	1.72	1.06–2.79	18	0.028
*CARD11*	0.58	0.35–0.95	38	0.033
*IGF1R*	0.47	0.23–0.94	22	0.034
*KMT2D*	0.62	0.4–0.96	47	0.034
*PTPRD*	0.66	0.46–0.96	62	0.029
*PTPRT*	0.71	0.52–0.97	85	0.029
*ROS1*	0.68	0.47–0.99	63	0.043
*TERT*	0.71	0.55–0.91	186	0.008

MSK-IMPACT, memorial sloan kettering cancer center integrated mutation profiling of actionable cancer targets; HR, hazard ratio; CI, confidence interval.

**FIGURE 1:**
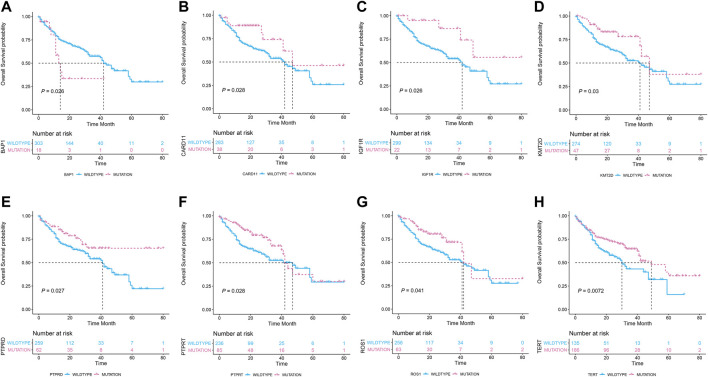
The association of the eight gene mutation statuses with the overall survival in 321 metastatic melanoma patients who received immune checkpoint inhibitor treatments. **(A)** Patients carrying *BP1* mutations (*n* = 18) had poor overall survival than the wildtype counterparts (*n* = 303). **(B–H)**: Patients carrying *CARD11, IGF1R, KMT2D, PTPRD, PTPRT, ROS1,* and *TERT* mutations had prolonged overall survival than their wildtype counterparts.

Kaplan–Meier survival curves are presented in Figure 1, and it was revealed that seven gene mutations were associated with better OS, except the *BAP1* gene mutation which was associated with poorer OS.

## Polygenic hazard score model was constructed and validated

Because the OS is also affected by the age, ICI regime, and TMB, we subsequently performed the multivariate Cox regression analysis to generate the adjusted HR for each of the eight gene mutations (Supplementary Table S3). Subsequently, PHS was calculated for each of 321 patients based on the genotype regarding the eight genes’ mutational status as illustrated in the method part. After that, univariate and multivariate Cox regression analyses were performed to test the PHS model. It was revealed that, PHS can effectively predict the OS in the MSK-IMPACT cohort (i.e., the discovery cohort, HR = 1·54, 95%CI, 1.25–1.95; *p* < 0.001) and this prediction was independent from the TMB, age, sex, and ICI regime (adjusted HR = 1.84, 95%CI, 1.22–2.79; *p* = 0.004). Details are included in Table 3.

**TABLE 3:** Univariate and multivariate analyses of PHS associated with overall survival in the MSK-IMPACT metastatic melanoma cohort that underwent ICI treatment.

Variables	Univariate analysis			Multivariate analysis		
	HR	95%CI	*p* value	HR	95%CI	*p* value
PHS	1.54	1.25–1.91	<0.001*	1.84	1.22–2.79	0.004*
Sex						
female	1		reference	1		reference
male	0.99	0.69–1.43	0.986	1.23	0.84–1.80	0.282
ICI regime						
PD-1/PD-L1	1		reference	1		reference
CTLA-4	0.61	0.38–0.97	0.039*	0.699	0.44–1.12	0.137
Combo	0.77	0.50–1.17	0.218	0.75	0.49–1.16	0.200
TMB	0.99	0.98–1.00	0.045*	1.01	1.00–1.02	0.164

PHS, polygenic hazard score; ICI, immune checkpoint inhibitors; MSK-IMPACT, memorial sloan kettering cancer center integrated mutation profiling of actionable cancer targets; HR, hazard ratio; CI, confidence interval; TMB, tumor mutation burden; Combo, anti CTLA-4, combined with anti PD-1/PD-L1; * The results were in bold if the *p*-value was less than 0.05.

**FIGURE 2:**
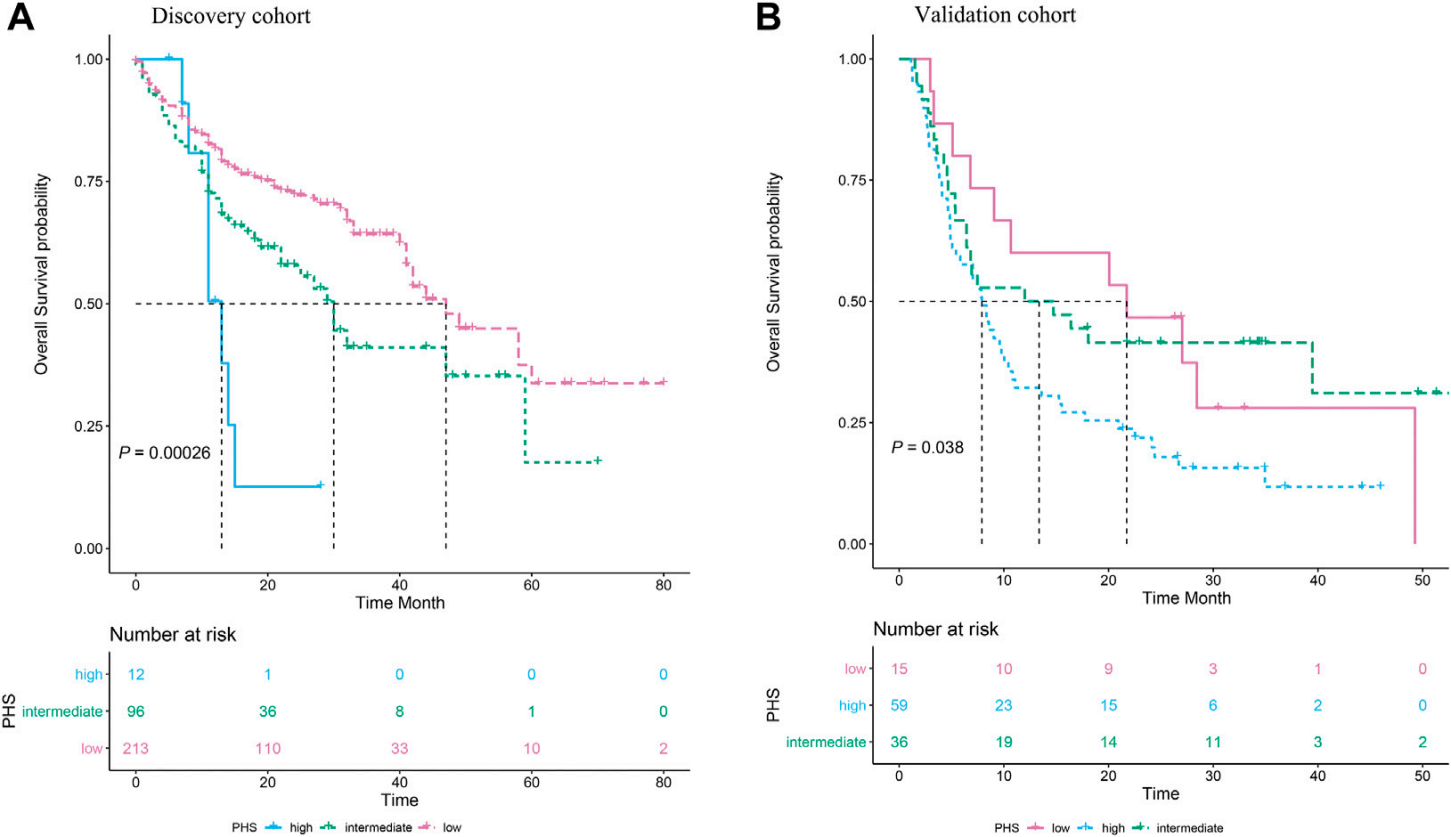
The association of polygenic hazard ratio (PHS) levels with the overall survival in metastatic melanoma patients treated with immune checkpoint inhibitors in two independent datasets. **(A)**. Three subgroups, i.e., the high PHS, the intermediate PHS, and the low PHS, have significantly different overall survival rates in the MSK-IMPACT cohort. **(B)**The PHS model was successfully validated in the DFCI metastatic melanoma cohort, the group with high PHS had the poorest overall survival compared to the groups with intermediate PHS and low PHS.

Additionally, the Kaplan–Meier survival curves of the three groups which were divided by the PHS value were presented in Figure 2A (log-Rank *p* < 0.001). In the DFCIMM cohort, the PHS was calculated using the same adjusted HR of those eight genes and was validated. It was shown that PHS was significantly associated with OS in 110 patients, the Kaplan–Meier survival curves of the three groups which were divided by PHS value are presented in Figure 2B (log-Rank *p* = 0.038).

### *CARD11* and *PTPRD* were associated with higher tumor-infiltrated immune cells in abundance

**FIGURE 3:**
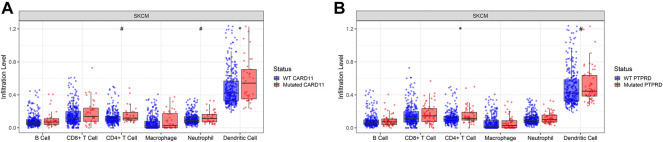
*CARD11* and *PTPRD* genes were associated with higher tumor-infiltrated immune cell abundance in skin melanoma tumor samples from The Cancer Genome Atlas database. **(A)**. *CARD11* gene mutational status was significantly associated with more dendric cells (*p* < 0.05), and also associated with more CD4+ T cells and neutrophilic cells (*p* < 0.1). **(B)**. *PTPRD* gene mutational status was significantly associated with more CD4+ T cells (*p* < 0.05), and was also associated with more dendric cells (*p* < 0.1). *: *p* < 0.05, #: *p* < 0.1.

In those eight genes whose mutations can impact the OS in the metastatic melanoma patients, two genes, *CARD11* and *PTPRD,* were associated with higher tumor-infiltrated immune cell abundance in skin melanoma tumor samples from the TCGA database. Boxplots in the Figure 3 showed that samples carrying *CARD11* or *PTPRD* mutations harbored more dendric cells and CD4+ T cells. Specifically, *CARD11* mutation was significantly associated with more dendric cells (*p* < 0.05); *PTPRD* mutation was significantly associated with more CD4+ T cells (*p* < 0.05).

## Discussion

For ICI therapies in the metastatic melanoma, which was widely recognized as an immunogenic malignancy, the outcomes were different from patient to patient, suggesting that good biomarkers were critically needed. For patients who could not benefit from the ICI therapy, ICI therapies might result with unnecessary immune-mediated toxicities, sometimes this adverse effect could be lethal (Weber et al., 2015).

In our research, we identified eight genes which were significantly associated with longer median OS in the metastatic melanoma. Previous reports also suggested that a higher burden of gene copy number loss was associated with the anti-CTLA-4 and anti-PD-1 therapy response and the *TP53* gene mutation was negatively associated with survival in the melanoma treated with anti-CTLA4 therapies (Van Allen et al., 2015; Weber et al., 2015; Li et al., 2017; Roh et al., 2017; Seibert et al., 2018; Xiao et al., 2018; Tan et al., 2019; Varga et al., 2019), However, a single gene prediction model was not effective enough to distinguish the responders or non-responders in ICI treatments.

Polygenic models have been well established in the risk prediction realm. But, for the melanoma or ICI therapies, there is no polygenic model for now. Hence, we developed this PHS model to predict the ICI therapy outcomes for the first time, and validated our PHS model in another independent cohort. Interestingly, when we performed the multivariate Cox regression model including the age, sex, ICI regimes, TMB, and PHS, it was found that PHS was still with a *p* value less than 0.05 while the *p* value of TMB changed into a larger value than 0.05, which suggested that PHS could more accurately and effectively predict the survival in metastatic melanoma under ICI treatments than TMB.

Furthermore, we found that two out of those eight gene (*CARD11* and *PTPRD*) mutations were associated with more tumor-infiltrated immune cells in tumor tissues. This can partially explain the different outcomes after ICI treatments, because it was reported that more intratumor immune cells may lead to the benefit for ICI therapies in melanoma (Siddiqui et al., 2019). However, the other six gene mutations were not significantly associated with different tumor-infiltrated immune cell abundance. As for the *CADR11* gene, researchers found that it played an important role in maintaining the normal adaptive immunity (Ma et al., 2017). Previous research also proved that deletion of the *CADR11* gene (alias *CARMA1*) in regulatory T cells could produce IFN-γ leading to dramatical anti-tumor effects, and contributed positively to the ICI therapies (Di Pilato et al., 2019). Another study reported that *CARD11* mutant skin cutaneous melanoma had higher immunogenicity compared to wildtype counterparts, and CARD11 mutation was associated with a longer OS after ICI therapy (Si et al., 2021). This was consistent with our findings, i.e., melanoma patients carrying mutational *CARD11* samples survived longer than the wildtype melanoma patients after ICI therapies. As for the *PTPRD* gene, there were no reports about this gene in the tumor immunity area yet, but the *PTPRD* gene was involved in the *JAK/STAT* signaling which was found to be associated with tumor immunotherapy (Owen et al., 2019). *PTPRD* encodes a kind of protein tyrosine phosphatase which is involved in tyrosine phosphorylation. Walia et al. reported that *PTPRD* acts as a tumor-suppressor and was one of the most frequently mutated genes in cutaneous metastatic melanoma, mutant PTPRD loses the function of inhibiting cell proliferation and migration, some kind of mutants even promote cell migration (Walia et al., 2014).

In conclusion, we built a PHS model which can effectively and independently predict the ICI outcome in metastatic melanoma, and we successfully validated our PHS model in an independent cohort. This PHS model may serve to help the clinical decision-making for metastatic melanoma patients. However, due to the relatively small patients’ size, this PHS model may need improvements when larger cohorts are available.

## Data Availability

The original contributions presented in the study are included in the article/Supplementary Material; further inquiries can be directed to the corresponding author.
