# Supplementary material for: Eight gene mutation-based polygenic hazard score as a potential predictor for immune checkpoint inhibitor therapy outcome in metastatic melanoma
Source: Front Mol Biosci. 2022 Sep 2;9:1001792. doi: 10.3389/fmolb.2022.1001792 (PMC9478752; doi:10.3389/fmolb.2022.1001792)
Supplement: Supplementary file 2 [file Table1.PDF]

List of genes/transcripts included on the MSK-IMPACT panel

Gene Name	Transcript ID
ABL1	NM_005157
ACVR1	NM_001111067
AGO2	NM_012154
AKT1	NM_001014431
AKT2	NM_001626
AKT3	NM_005465
ALK	NM_004304
ALOX12B	NM_001139
AMER1	NM_152424
ANKRD11	NM_013275
APC	NM_000038
AR	NM_000044
ARAF	NM_001654
ARID1A	NM_006015
ARID1B	NM_020732
ARID2	NM_152641
ARID5B	NM_032199
ASXL1	NM_015338
ASXL2	NM_018263
ATM	NM_000051
ATR	NM_001184
ATRX	NM_000489
AURKA	NM_003600
AURKB	NM_004217
AXIN1	NM_003502
AXIN2	NM_004655
AXL	NM_021913
B2M	NM_004048
BABAM1	NM_001033549
BAP1	NM_004656
BARD1	NM_000465
BBC3	NM_001127240
BCL10	NM_003921
BCL2	NM_000633
BCL2L1	NM_138578
BCL2L11	NM_138621
BCL6	NM_001706
BCOR	NM_001123385
BIRC3	NM_182962
BLM	NM_000057
BMPR1A	NM_004329
BRAF	NM_004333
BRCA1	NM_007294
BRCA2	NM_000059
BRD4	NM_058243
BRIP1	NM_032043
BTK	NM_000061
CALR	NM_004343
CARD11	NM_032415
CARM1	NM_199141
CASP8	NM_001080125
CBFB	NM_022845
CBL	NM_005188
CCND1	NM_053056
CCND2	NM_001759
CCND3	NM_001760
CCNE1	NM_001238
CD274	NM_014143
CD276	NM_001024736
CD79A	NM_001783
CD79B	NM_001039933
CDC42	NM_001791
CDC73	NM_024529

CDH1	NM_004360
CDK12	NM_016507
CDK4	NM_000075
CDK6	NM_001145306
CDK8	NM_001260
CDKN1A	NM_078467
CDKN1B	NM_004064
CDKN2Ap14ARF	NM_058195
CDKN2Ap16INK4A	NM_000077
CDKN2B	NM_004936
CDKN2C	NM_078626
CEBPA	NM_004364
CENPA	NM_001809
CHEK1	NM_001274
CHEK2	NM_007194
CIC	NM_015125
CREBBP	NM_004380
CRKL	NM_005207
CRLF2	NM_022148
CSDE1	NM_001242891
CSF1R	NM_005211
CSF3R	NM_000760
CTCF	NM_006565
CTLA4	NM_005214
CTNNB1	NM_001904
CUL3	NM_003590
CXCR4	NM_003467
CYLD	NM_001042355
CYSLTR2	NM_020377
DAXX	NM_001141970
DCUN1D1	NM_020640
DDR2	NM_006182
DICER1	NM_030621
DIS3	NM_014953
DNAJB1	NM_006145
DNMT1	NM_001379
DNMT3A	NM_022552
DNMT3B	NM_006892
DOT1L	NM_032482
DROSHA	NM_013235
DUSP4	NM_001394
E2F3	NM_001949
EED	NM_003797
EGFL7	NM_201446
EGFR	NM_005228
EIF1AX	NM_001412
EIF4A2	NM_001967
EIF4E	NM_001130678
ELF3	NM_004433
EP300	NM_001429
EPAS1	NM_001430
EPCAM	NM_002354
EPHA3	NM_005233
EPHA5	NM_004439
EPHA7	NM_004440
EPHB1	NM_004441
ERBB2	NM_004448
ERBB3	NM_001982
ERBB4	NM_005235
ERCC2	NM_000400
ERCC3	NM_000122
ERCC4	NM_005236
ERCC5	NM_000123
ERF	NM_006494
ERG	NM_182918
ERRFI1	NM_018948

ESR1	NM_001122740
ETV1	NM_001163147
ETV6	NM_001987
EZH1	NM_001991
EZH2	NM_004456
FAM175A	NM_139076
FAM46C	NM_017709
FAM58A	NM_152274
FANCA	NM_000135
FANCC	NM_000136
FAT1	NM_005245
FBXW7	NM_033632
FGF19	NM_005117
FGF3	NM_005247
FGF4	NM_002007
FGFR1	NM_001174067
FGFR2	NM_000141
FGFR3	NM_000142
FGFR4	NM_213647
FH	NM_000143
FLCN	NM_144997
FLT1	NM_002019
FLT3	NM_004119
FLT4	NM_182925
FOXA1	NM_004496
FOXL2	NM_023067
FOXO1	NM_002015
FOXP1	NM_001244814
FUBP1	NM_003902
FYN	NM_153047
GATA1	NM_002049
GATA2	NM_032638
GATA3	NM_002051
GLI1	NM_005269
GNA11	NM_002067
GNAQ	NM_002072
GNAS	NM_000516
GPS2	NM_004489
GREM1	NM_013372
GRIN2A	NM_001134407
GSK3B	NM_002093
H3F3A	NM_002107
H3F3B	NM_005324
H3F3C	NM_001013699
HGF	NM_000601
HIST1H1C	NM_005319
HIST1H2BD	NM_021063
HIST1H3A	NM_003529
HIST1H3B	NM_003537
HIST1H3C	NM_003531
HIST1H3D	NM_003530
HIST1H3E	NM_003532
HIST1H3F	NM_021018
HIST1H3G	NM_003534
HIST1H3H	NM_003536
HIST1H3I	NM_003533
HIST1H3J	NM_003535
HIST2H3C	NM_021059
HIST2H3D	NM_001123375
HIST3H3	NM_003493
HLA-A	NM_001242758
HLA-B	NM_005514
HNF1A	NM_000545
HOXB13	NM_006361
HRAS	NM_001130442
ICOSLG	NM_015259

ID3	NM_002167
IDH1	NM_005896
IDH2	NM_002168
IFNGR1	NM_000416
IGF1	NM_001111285
IGF1R	NM_000875
IGF2	NM_001127598
IKBKE	NM_014002
IKZF1	NM_006060
IL10	NM_000572
IL7R	NM_002185
INHA	NM_002191
INHBA	NM_002192
INPP4A	NM_001134224
INPP4B	NM_001101669
INPPL1	NM_001567
INSR	NM_000208
IRF4	NM_002460
IRS1	NM_005544
IRS2	NM_003749
JAK1	NM_002227
JAK2	NM_004972
JAK3	NM_000215
JUN	NM_002228
KDM5A	NM_001042603
KDM5C	NM_004187
KDM6A	NM_021140
KDR	NM_002253
KEAP1	NM_203500
KIT	NM_000222
KLF4	NM_004235
KMT2A	NM_001197104
KMT2B	NM_014727
KMT2C	NM_170606
KMT2D	NM_003482
KNSTRN	NM_033286
KRAS	NM_033360
LATS1	NM_004690
LATS2	NM_014572
LMO1	NM_002315
LYN	NM_002350
MALT1	NM_006785
MAP2K1	NM_002755
MAP2K2	NM_030662
MAP2K4	NM_003010
MAP3K1	NM_005921
MAP3K13	NM_004721
MAP3K14	NM_003954
MAPK1	NM_002745
MAPK3	NM_002746
MAPKAP1	NM_001006617
MAX	NM_002382
MCL1	NM_021960
MDC1	NM_014641
MDM2	NM_002392
MDM4	NM_002393
MED12	NM_005120
MEF2B	NM_001145785
MEN1	NM_000244
MET	NM_000245
MGA	NM_001164273
MITF	NM_198159
MLH1	NM_000249
MPL	NM_005373
MRE11A	NM_005591
MSH2	NM_000251

MSH3	NM_002439
MSH6	NM_000179
MSI1	NM_002442
MSI2	NM_138962
MST1	NM_020998
MST1R	NM_002447
MTOR	NM_004958
MUTYH	NM_001128425
MYC	NM_002467
MYCL1	NM_001033082
MYCN	NM_005378
MYD88	NM_002468
MYOD1	NM_002478
NBN	NM_002485
NCOA3	NM_181659
NCOR1	NM_006311
NEGR1	NM_173808
NF1	NM_001042492
NF2	NM_000268
NFE2L2	NM_006164
NFKBIA	NM_020529
NKX2-1	NM_001079668
NKX3-1	NM_006167
NOTCH1	NM_017617
NOTCH2	NM_024408
NOTCH3	NM_000435
NOTCH4	NM_004557
NPM1	NM_002520
NRAS	NM_002524
NSD1	NM_022455
NTHL1	NM_002528
NTRK1	NM_002529
NTRK2	NM_006180
NTRK3	NM_001012338
NUF2	NM_031423
NUP93	NM_014669
PAK1	NM_002576
PAK7	NM_177990
PALB2	NM_024675
PARK2	NM_004562
PARP1	NM_001618
PAX5	NM_016734
PBRM1	NM_018313
PDCD1	NM_005018
PDCD1LG2	NM_025239
PDGFRA	NM_006206
PDGFRB	NM_002609
PDPK1	NM_002613
PGR	NM_000926
PHOX2B	NM_003924
PIK3C2G	NM_004570
PIK3C3	NM_002647
PIK3CA	NM_006218
PIK3CB	NM_006219
PIK3CD	NM_005026
PIK3CG	NM_002649
PIK3R1	NM_181523
PIK3R2	NM_005027
PIK3R3	NM_003629
PIM1	NM_002648
PLCG2	NM_002661
PLK2	NM_006622
PMAIP1	NM_021127
PMS1	NM_000534
PMS2	NM_000535
PNRC1	NM_006813

POLD1	NM_002691
POLE	NM_006231
PPARG	NM_015869
PPM1D	NM_003620
PPP2R1A	NM_014225
PPP4R2	NM_174907
PPP6C	NM_002721
PRDM1	NM_001198
PRDM14	NM_024504
PREX2	NM_024870
PRKAR1A	NM_212471
PRKCI	NM_002740
PRKD1	NM_002742
PTCH1	NM_000264
PTEN	NM_000314
PTP4A1	NM_003463
PTPN11	NM_002834
PTPRD	NM_002839
PTPRS	NM_002850
PTPRT	NM_133170
RAB35	NM_006861
RAC1	NM_018890
RAC2	NM_002872
RAD21	NM_006265
RAD50	NM_005732
RAD51	NM_002875
RAD51B	NM_133509
RAD51C	NM_058216
RAD51D	NM_133629
RAD52	NM_134424
RAD54L	NM_001142548
RAF1	NM_002880
RARA	NM_000964
RASA1	NM_002890
RB1	NM_000321
RBM10	NM_001204468
RECQL	NM_032941
RECQL4	NM_004260
REL	NM_002908
RET	NM_020975
RFWD2	NM_022457
RHEB	NM_005614
RHOA	NM_001664
RICTOR	NM_152756
RIT1	NM_006912
RNF43	NM_017763
ROS1	NM_002944
RPS6KA4	NM_003942
RPS6KB2	NM_003952
RPTOR	NM_020761
RRAGC	NM_022157
RRAS	NM_006270
RRAS2	NM_012250
RTEL1	NM_032957
RUNX1	NM_001754
RXRA	NM_002957
RYBP	NM_012234
SDHA	NM_004168
SDHAF2	NM_017841
SDHB	NM_003000
SDHC	NM_003001
SDHD	NM_003002
SESN1	NM_014454
SESN2	NM_031459
SESN3	NM_144665
SETD2	NM_014159

SETD8	NM_020382
SF3B1	NM_012433
SH2B3	NM_005475
SH2D1A	NM_002351
SHOC2	NM_007373
SHQ1	NM_018130
SLX4	NM_032444
SMAD2	NM_001003652
SMAD3	NM_005902
SMAD4	NM_005359
SMARCA4	NM_003072
SMARCB1	NM_003073
SMARCD1	NM_003076
SMO	NM_005631
SMYD3	NM_001167740
SOCS1	NM_003745
SOS1	NM_005633
SOX17	NM_022454
SOX2	NM_003106
SOX9	NM_000346
SPEN	NM_015001
SPOP	NM_001007228
SPRED1	NM_152594
SRC	NM_198291
SRSF2	NM_003016
STAG2	NM_001042749
STAT3	NM_139276
STAT5A	NM_003152
STAT5B	NM_012448
STK11	NM_000455
STK19	NM_004197
STK40	NM_032017
SUFU	NM_016169
SUZ12	NM_015355
SYK	NM_003177
TAP1	NM_000593
TAP2	NM_018833
TBX3	NM_016569
TCEB1	NM_005648
TCF3	NM_001136139
TCF7L2	NM_001146274
TEK	NM_000459
TERT	NM_198253
TET1	NM_030625
TET2	NM_001127208
TGFBR1	NM_004612
TGFBR2	NM_001024847
TMEM127	NM_001193304
TMPRSS2	NM_001135099
TNFAIP3	NM_006290
TNFRSF14	NM_003820
TOP1	NM_003286
TP53	NM_000546
TP53BP1	NM_001141980
TP63	NM_003722
TRAF2	NM_021138
TRAF7	NM_032271
TSC1	NM_000368
TSC2	NM_000548
TSHR	NM_000369
U2AF1	NM_006758
UPF1	NM_002911
VEGFA	NM_001171623
VHL	NM_000551
VTCN1	NM_024626
WHSC1	NM_001042424

WHSC1L1	NM_023034
WT1	NM_024426
WWTR1	NM_001168280
XIAP	NM_001167
XPO1	NM_003400
XRCC2	NM_005431
YAP1	NM_001130145
YES1	NM_005433
ZFHX3	NM_006885
