# Supplementary material for: Eight gene mutation-based polygenic hazard score as a potential predictor for immune checkpoint inhibitor therapy outcome in metastatic melanoma
Source: Front Mol Biosci. 2022 Sep 2;9:1001792. doi: 10.3389/fmolb.2022.1001792 (PMC9478752; doi:10.3389/fmolb.2022.1001792)
Supplement: Supplementary file 3 [file Table3.DOCX]

| **Supplementary table 3**: Hazard ratio was derived after adjusted with age, sex, ICI regime and TMB | | |
| --- | --- | --- |
| Gene Symbol | HR | Log HR |
| *BAP1* | 1.85 | 0.887525271 |
| *CARD11* | 0.44 | -1.184424571 |
| *IGF1R* | 0.45 | -1.152003093 |
| *KMT2D* | 0.7 | -0.514573173 |
| *PTPRD* | 0.73 | -0.454031631 |
| *PTPRT* | 0.67 | -0.577766999 |
| *ROS1* | 0.7 | -0.514573173 |
| *TERT* | 0.71 | -0.49410907 |
| Abbreviations: HR, hazard ratio; CI, confident interval; TMB, tumor mutation burden; log HR, log hazard radio. | | |
